# Feeding *Drosophila* highly radioresistant fungi improves survival and gut morphology following acute gamma radiation exposure

**DOI:** 10.1038/s41598-025-31545-6

**Published:** 2025-12-14

**Authors:** Robert P. Volpe, Hye Jin Hwang, Rachel T. Cox

**Affiliations:** 1 Department of Biochemistry and Molecular Biology, Uniformed Services University, Bethesda, MD 20814 USA; 2 Henry M. Jackson Foundation for the Advancement of Military Medicine, Bethesda, MD 20817 USA

**Keywords:** Fungi, Radioresistant, Irradiation, Lifespan, Gut, *Drosophila*, Cancer, Microbiology, Zoology

## Abstract

Diverse fungi have been historically vital reservoirs of drug discovery, providing life-saving pharmaceuticals. Many species of fungi, yeasts in particular, are highly resistant to radiation, with their cellular contents potentially conferring dietary radioresistance. We developed a *Drosophila* model to test whether feeding two highly radioresistant fungi, *Aureobasidium pullulans* and *Rhodotorula taiwanensis,* could improve fly lifespan and gut morphology after acute irradiation. We constructed a dosimetry curve for the lifespan response of males and females to irradiation and found dose-dependent and sex-specific effects on lifespan. We also determined that the sex-specific response to irradiation correlated with nuclear morphology defects in the gut, with the more radiosensitive males displaying increased midgut cellular holes and aberrant nuclear morphology. To determine if feeding *Aureobasidium pullulans* and *Rhodotorula taiwanensis* before irradiation could improve survival and gut morphology, we first exclusively fed males and females each fungus and observed that they tolerated the diet well. Using these methods, we found that only two days of pre-feeding *Aureobasidium pullulans* increased male lifespan, but not female, after irradiation, and improved nuclear morphology in the gut. However, dietary *Rhodotorula taiwanensis* was not protective. Overall, this study identified a highly radioresistant dietary fungus, *Aureobasidium pullulans*, as effective for extending male *Drosophila* lifespan and improving gut morphology following irradiation. Since the gut is particularly sensitive to the effects of irradiation, this fungus indicates a potential therapeutic for patients undergoing radiotherapy. Furthermore, this method could identify additional radioresistant fungi that protect the gut from radiation injury.

**Supplementary Information:**

The online version contains supplementary material available at 10.1038/s41598-025-31545-6.

## Introduction

Fungi synthesize a large number of naturally occurring bioactive molecules from which researchers have identified a wide array of vital pharmaceuticals such as antibiotics, immunosuppressants, and cholesterol-lowering drugs^1,2^. Within this large and diverse kingdom are species that are highly radiation-resistant, with fungal species growing in the reactor of the damaged Chernobyl Nuclear Power Plant among the first ones identified^3^. Since then, many species of fungi have been shown to survive acute and chronic exposures to astoundingly high levels of ionizing radiation (IR), far beyond any that are naturally occurring^4,5^. Several mechanisms contribute to the astonishing ability of these microbes to grow and reproduce in highly irradiated environments. Some radiation-resistant organisms accumulate antioxidant compounds such as manganous peptide phosphate complexes to survive the oxidative stress of IR^6,7^. Melanin often accumulates in response to stress in various fungi, and its chemical properties include broad optical absorption and the ability to interact with ionizing radiation, lending it antioxidant activity^3,8,9^. Given the diversity of the kingdom and the fact that so many species demonstrate radioresistance to levels of IR not found in nature, this suggests that additional metabolites and molecules conferring radioresistance have yet to be identified.

*Aureobasidium pullulans*, a dimorphic ascomycetous yeast, grows on the inner parts of the damaged Chernobyl Nuclear Power Plant and the International Space Station, and is considered a polyextremotolerant fungus^3,10,11^. *Aureobasidium pullulans* (*A. pullulans*) is usually light in color but becomes darker under stress due to melanin production, which is thought to confer enhanced survival^10,12^. *Rhodotorula taiwanensis* (*R. taiwanensis*), a basidiomycetous carotenogenic yeast, was characterized for its ability to tolerate high levels of chronic radiation and low pH as part of an initiative to identify fungi that could be harnessed for the bioremediation of radioactive waste sites^5^. *R. taiwanensis* can grow under chronic 66 Gy/h gamma radiation at pH 2.3, giving this fungus remarkable resiliency^5^. Since *R. taiwanensis* and *A. pullulans* survive IR in the absence of melanin, there are likely unidentified metabolites and small molecules that function to protect them against the damaging effects of IR.

IR damages DNA by causing double-strand breaks (DSBs), lipids, such as those in membranes, through peroxidation and proteins through various modifications^13^. It is generally accepted that DNA is most sensitive to IR since the DSBs are caused directly by the radiation exposure. For example, the effects of cytoplasmic irradiation can cause nuclear mutations and DSBs while being minimally cytotoxic^14,15^. In addition, studies have indicated that increased amounts of DNA in the nucleus make cells more susceptible to DSBs^16^. Thus, human mitotic cells, such as in the bone marrow, gut and reproductive organs, are highly sensitive to the effects of IR exposure^17^. In people, acute radiation syndrome (ARS) results from exposures as low as 1 Gy, with the hematopoietic system, the gastrointestinal tract, and cerebrovascular system becoming damaged with increasing amounts of IR^18^.

Using *Drosophila*, we investigated whether ingestion of *A. pullulans* and *R. taiwanensis* could provide radioprotection to the gut and extend lifespan. *Drosophila* have a rapid lifecycle, short lifespan, and large genetic toolkit, making them an ideal animal in which to test for dietary protection against IR. Furthermore, *Drosophila* have organ systems that are highly homologous to human physiology, sufficient for modeling numerous human diseases^19,20^. For example, the adult gut exhibits clear regional differences in morphology, physiology, and gene expression along its anterior–posterior axis and has emerged as an important model to study epithelial damage and intestinal stem cell biology^21–26^. DNA damage and DSBs also occur in the fly gut after exposure to IR^27,28^. *Drosophila* are natural fungivores and readily consume diverse yeast species with notable effects on physiology and behavior^29,30^. Thus, natural feeding would allow direct contact between the radioresistant fungi and highly radiosensitive gut cells, optimizing any potential radioprotective benefits.

To test the protective effects of dietary *A. pullulans* and *R. taiwanensis* after acute exposure to gamma radiation, we first constructed radiation survival curves for male and female lifespans to identify doses that were not acutely detrimental to fly health, yet high enough to make lifespan measurements manageable. Males were more sensitive to the effects of IR compared to females, as expected^31–33^. In addition, we observed that male gut morphology was significantly more disrupted compared to females receiving the same IR dose. Next, we fed males and females exclusive diets of both fungi and determined that, although they shortened lifespan, neither was acutely toxic, nor did they have detrimental effects on development. To test for dietary protection, we fed flies either fungus for two days before IR exposure and determined that *A. pullulans*, but not *R. taiwanensis*, enhanced radiation survival of males, but not females, for the next fifteen to twenty days. Males fed *A. pullulans* and subsequently exposed to IR showed improved nuclear morphology in the gut compared to non-fed control males, suggesting this could be a contributing factor to the increased survival. Overall, this study demonstrates that *Drosophila* is an effective model to successfully identify highly radioresistant dietary fungi that confer radioresistance to the host. Notably, we identified *A. pullulans* as a promising radioprotectant, which could be further tested in higher eukaryotes.

## Materials and methods

### Drosophila and fungal strains

Fly strains used in this study: *w*^*1118*^ and *catalase*^*n1*^/*TM3, Sb*^*1*^*, Ser* (Kyoto *Drosophila* Stock Center, #107554). Fly stocks were maintained in standard cornmeal fly food vials at room temperature and a 12-h light cycle. The strain of *Aureobasidium pullulans var. namibiae* (*A. pullulans*) (EXF-1147) used in this study was obtained from Microbial Culture Collection EX curated by the Biotechnical Faculty of the University of Ljubljana in Ljubljana, Slovenia. The strain of *Rhodotorula taiwanensis* (*R. taiwanensis*) (MD-1149) was isolated from an acid mine drainage facility in Allegany County, Maryland, USA, and curated in the collection of Dr. Michael Daly of the Department of Pathology of the Uniformed Services University of the Health Sciences in Bethesda, Maryland, USA. Dietary yeast cultures were grown at room temperature on standard YPD agar plates [1% Bacto Yeast Extract (Cat# 212750, Thermo Fisher Scientific, Waltham, MA, USA), 2% Peptone (Cat# 211677, Thermo Fisher Scientific, Waltham, MA, USA), 2% Dextrose (Cat# PHR1000, Millipore Sigma, St. Louis, MO, USA), 2% Bacto Agar (Cat# DF-0140-01-0, Thermo Fisher Scientific, Waltham, MA, USA)] covered with gamma-sterilized dialysis membrane (Frey Scientific Dialysis Tubing, Cat# 1591654, Dialysis Tubing, Frey Scientific®, Greenville, WI, USA) to ensure only yeast was removed during collection. For pre-feeding IR exposured cultures, *A. pullulans* and *R. taiwanensis* were grown for 4 days and 1 day, respectively, under chronic irradiation at 35 Gy/h in a ^137^Cs-sourced gamma irradiator (Shepard and Associates 68A Mark1, San Fernando, CA, USA). Unirradiated fungi cultures were grown in the same location outside the irradiator to ensure both cultures grew under otherwise identical conditions. Acute radiation tolerances (D_10_) for both strains were determined by incremental irradiation of liquid YPD culture (OD_600_ = 0.8) in a ^60^Co-sourced gamma irradiator (Shepard and Associates, San Fernando, CA, USA) followed by colony forming unit (CFU) assay on solid YPD medium as previously described^34^. To achieve a bisecting exposure of *A. pullulans* culture, we cast a cylindrical radiation shield of pure lead (Rotometals, Inc., San Leandro, CA, USA) with a central cavity of approximately 65 mm × 30 mm × 12 mm to snugly fit a standard 60 mm petri dish. *A. pullulans* was streaked on solid YPD agar plate, placed in the radiation shield leaving half the plate exposed, and irradiated at ~ 35 Gy/h for 24 h in a ^137^Cs-sourced irradiator. The partially irradiated culture was then allowed to recover for 3 days. To observe phenotypic switching of melanin expression in response to cyclical irradiation, 20 µL of *A. pullulans* in liquid YPD (OD_600_ 0.8) was aliquoted to the center of a YPD agar plate. After one day of growth, the culture was exposed to alternating cycles of chronic radiation at 35 Gy/hr for 2 days followed by 2 days without exposure. To visualize accumulation of melanin in the cell walls of *A. pullulans* hyphae, cultures were imaged using a Motic BA210E (Motic, Universal City, TX, USA) at 400x (40 × objective x 10 × eyepiece).

### Fungal feeding

For administration of fungal diets, flies were housed in feeding chambers made from agar feeding plates encapsulated by perforated plastic beakers. Aliquots of fungi were carefully harvested with a silicone spatula from 2–3 day-old cultures grown on YPD plates and added to agar plates. Newly eclosed flies age 0–2 h old were collected and allowed to feed for two days, irradiated, then transferred to standard food vials supplemented with a small amount of live dry yeast (*Saccharomyces cerevisiae* (*S. cerevisiae*) (Red Star Active Dry Yeast, Red Star Yeast Company LLC, Milwaukee, WI, United States)). For determining the toxicity of, and survivorship on, *A. pullulans* and *R. taiwanensis* diets, twenty males or females, in triplicate, were continuously fed fungal paste for the entire lifespan, changing the plate and fungal paste every 1–2 days. A paste of water and *S. cerevisiae* was used as a control. To determine pupation and eclosion rates, twenty first instar larvae collected one day after egg laying were transferred into agar plates supplemented with *A. pullulans*, *R. taiwanensis* or a paste of control *S. cerevisiae* and grown at room temperature. The number of pupae was counted every 24 h after the onset of pupation. The number of eclosed adults was counted each day after the onset of eclosion. Each experiment was performed in triplicate. To visualize consumption of the fungi, representative flies and larvae were imaged using an Accu-scope 3076 digital microscope 0.67x-4.5x (Accu-Scope, Commack, NY, USA).

### Irradiation, dosimetry, and lifespan analysis

Flies were exposed to gamma radiation in standard polystyrene *Drosophila* vials (Genesee Scientific, Cat# 32-109, Morrisville, NC, USA) capped with cellulose-acetate stoppers (Genesee Scientific, Cat# 49-102, Morrisville, NC, USA) containing standard cornmeal fly media (8.25% corn syrup (Karo Light Corn Syrup, ACH Food Companies, Oakbrook Terrace, IL, United States), 5% corn meal (Cat# 62-100, Genesee Scientific, El Cajon, CA, United States), 1.25% dry inactivated yeast (Cat# 62-103, Genesee Scientific, El Cajon, CA, United States), 0.75% soy flour (Cat# 62-115, Genesee Scientific, El Cajon, CA, United States), 0.5% propionic acid (Cat# P1386, Millipore Sigma, St. Louis, MO, United States), 0.1% Tegosept (Ca# 20-258, Genesee Scientific, El Cajon, CA, United States) with 0.04 g live dry yeast (Red Star Active Dry Yeast, Red Star Yeast Company LLC, Milwaukee, WI, United States)). Six vials, two sets of matching triplicates, were irradiated per radiation treatment using a vendor-calibrated, ^137^Cs-sourced gamma irradiator. Vials were placed on a rotating platform to ensure uniform exposure of all samples to the source emission. Consistent and precise dosimetry for all sample irradiations was achieved by calculating the length of each exposure, accounting for daily source decay using the standard decay equation. Exposures were delivered at a dose rate of ~ 12.62 Gy/min. For lifespan analysis, twenty male and female adults were monitored daily after irradiation for their full lifespan, which was performed minimally in triplicate as described in *Fungal feeding* above. Since irradiation significantly weakens the animals, the determination of death for each fly was made by observing whether it failed to respond repeatedly to gentle probing, as indicated by head twitching and/or leg movements. To perform statistical analysis, the average daily survivorship data from the triplicates were compiled and entered into the Online Application for Survival Analysis 2 (OASIS) (https://sbi.postech.ac.kr/oasis2/)^35^. Statistical significance between control and experimental conditions was determined in OASIS using the Wilcoxon-Breslow-Gehan test (Supplementary Table 1).

### Immunostaining and gut injury analysis

Post-irradiated 4-day-old adult flies were dissected and immunostained as previously described, with slight modifications^36^. Briefly, 2-day-old adult flies were irradiated and subsequently maintained in standard food vials for an additional two days. The guts were dissected in 1 × phosphate-buffered saline (PBS) and fixed with 4% formaldehyde in 1 × PBS for thirty minutes at room temperature. After washing with Antibody wash solution (AWS, 0.1% TritonX-100, and 1% BSA in PBS) three times for twenty minutes, the tissues were stained with Alexa Fluor™ 488 Phalloidin (Cat# A12379, Invitrogen, Waltham, MA, USA) overnight at 4 °C. Following washes with AWS twice for 20 min, tissues were stained with 4′,6-Diamidino-2-phenylindole (DAPI) for 10 min at room temperature and mounted in Vectashield Antifade Mounting Medium (Cat# H-1000, Vector Laboratories, Newark, CA, USA). Images were obtained using a Zeiss LSM 980 confocal laser scanning microscope with a 63 × objective lens (Carl Zeiss Microscopy LLC, White Plains, NY, USA). The R4 region of the midgut was chosen, and a z-stacked image was obtained for the whole layer of enterocytes of randomly selected regions in R4, from just beneath the muscle cells to the lumen. The total number of guts analyzed is indicated in each figure legend. Quantification of abnormal nuclear shape, disrupted cellular barriers, and holes in the actin filament layer of enterocytes was determined by subjective measurement. Abnormal nuclear shape was determined if a nucleus had a smaller size relative to control and decreased DAPI staining in the nucleus. Disrupted cellular barriers between enterocytes were evaluated by loss of actin and abnormal cellular shapes, as demonstrated by the actin staining. We did not include cells which had lost actin labeling and had fainter DAPI staining in the category of disrupted cellular barriers since this phenotype was found in control non-irradiated animals. Holes in an actin filament layer were counted if the following conditions were satisfied: at least five clear round-shaped holes with a diameter of more than 1.5 µm in an actin layer within a single cell, or two holes with a diameter of more than 2.5 µm. For nuclei, the percentage of abnormal nuclei relative to total nuclei within each image of the guts was calculated and plotted in a graph generated using GraphPad Prism (GraphPad Software, version 10.4.2, Boston, Massachusetts USA, www.graphpad.com). For disruption to cellular barriers and holes in the actin filament later, the percentage of animals with gut injury in each replicate was calculated, averaged, and plotted in a graph generated using GraphPad Prism (GraphPad Software, version 10.4.2, Boston, Massachusetts USA, www.graphpad.com). A summary table of the image analyses is in Supplemental Spreadsheet 1. Significant differences between groups were tested as described in *Statistical analyses* below.

### Statistical analyses

To quantify the relationship between radiation dose and mortality, linear regression was conducted in Microsoft Excel (version 16.94.1), and the coefficient of determination (R^2^) was reported as a measure of goodness-of-fit. Lifespan data were analyzed using the OASIS 2 online platform for survival analysis (^35^; https://sbi.postech.ac.kr/oasis2/) using compiled average of three triplicates. Mean lifespan statistical significance between groups was determined using log-rank and weighted log-rank (Wilcoxon–Breslow-Gehan) test^37^. The analysis of gut injury was performed using GraphPad Prism (GraphPad Software, version 10.4.2, Boston, Massachusetts USA, www.graphpad.com). Two-way analysis of variance (ANOVA) with multiple comparisons followed by Tukey’s post hoc test was performed to evaluate the effect of sex (male, female) and irradiation (− IR, + IR) on abnormal physiological events. Mean lifespans, standard errors, and Bonferroni p-values were reported to assess the impact of dietary intervention on survival following irradiation and are presented in Supplementary Table 1.

### Biological, chemical, and radiological safety

All experimental work was performed in strict adherence to the biological, chemical, and radiological safety protocols and regulations established by the Uniformed Services University of the Health Sciences and its Environmental Health and Radiation Safety Divisions.

## Results

### *Drosophila* exhibit dose-dependent lifespan sensitivity to exposure to acute irradiation

**Fig. 1:**
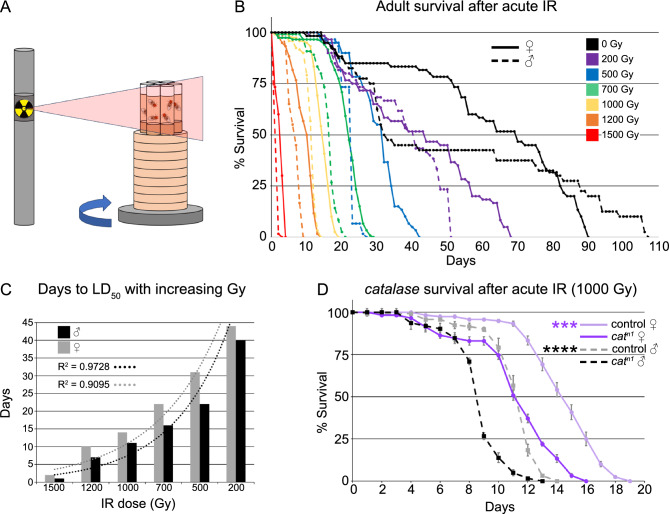
Dosimetry and radiation sensitivity of *Drosophila*. (**A**) Schematic of *Drosophila* irradiation sequence. (**B**) Lifespan of male and female flies acutely exposed to 0–1500 Gy gamma radiation. Radiation survival negatively correlates with dose. Males (dashed lines) were more sensitive than females (solid line) for all doses. (**C**) Graph showing a negative exponential relationship between the days to LD_50_ for males and females and increasing IR dose, indicated by R^2^ close to 1.0 for both sexes. (**D**) Lifespan of control (*w*^*1118*^) and *catalase*^*n1*^ (*cat*^*n1*^) mutants after 1000 Gy acute irradiation. *cat*^*n1*^ null flies are more sensitive to irradiation than control, and males are more sensitive than females for both genotypes. IR = ionizing radiation. Graphs were plotted using Microsoft Excel. Each point on the lifespan represents the average of triplicates of twenty flies that were irradiated simultaneously. Significance: (D) p = 0.0002 (***) for females and 0.000049 (****) for males.

In order to use *Drosophila* as a model to test dietary radioprophylactic properties of highly radioresistant fungi, we designed a protocol for irradiating adult flies and determined lifespan dose curves for a range of radiation exposures. For consistent irradiation, six standard fly food vials were elevated to the appropriate height on a rotating platform in a ^137^Cs-source irradiator (Fig. 1A). A blank vial was placed in the middle of the vial cluster. This configuration ensured that all flies were exposed to the correct dose of irradiation. This also enabled us to simultaneously test experimental and control groups in triplicate. We tested the effect of 0–1500 Gy gamma radiation on the lifespan of *w*^*1118*^ male and female adult flies (Fig. 1B). The normal (*w*^*1118*^) *Drosophila* lifespan is approximately 100 days (Fig. 1B, black lines). Twenty newly eclosed males and females, in triplicate, were simultaneously exposed to increasing doses of acute gamma radiation (Fig. 1B). Exposed males (Fig. 1B, dashed lines) were more sensitive to the effect of IR compared to females (Fig. 1B, solid lines) for every dose. In addition, the exponential decline in the number of days to reach LD_50_ of males and females was observed with increased IR dose, with strong coefficient of determination (R^2^) values of 0.9728 and 0.9095 for males and females, respectively (Fig. 1C). Finally, as a positive control, we tested whether *Drosophila* mutants lacking the free-radical scavenging enzyme *catalase* (*cat*) are more sensitive to IR exposure using our method. *cat* null flies normally exhibit wild type lifespans but are sensitive to oxidative stress^38,39^. Two-day-old adult *cat*^*n1*^ null mutants exposed to 1000 Gy had shorter lifespans compared to *w*^*1118*^, with males more sensitive than females (Fig. 1D, p = 0.0002 (***) (female) and p = 0.000049 (****) (male)). Together, these data demonstrate that our method exposing male and female *Drosophila* to IR consistently shortened lifespan in a dose-sensitive and sex-specific manner.

### *Drosophila* male guts are more sensitive to the effects of irradiation compared to females

**Fig. 2:**
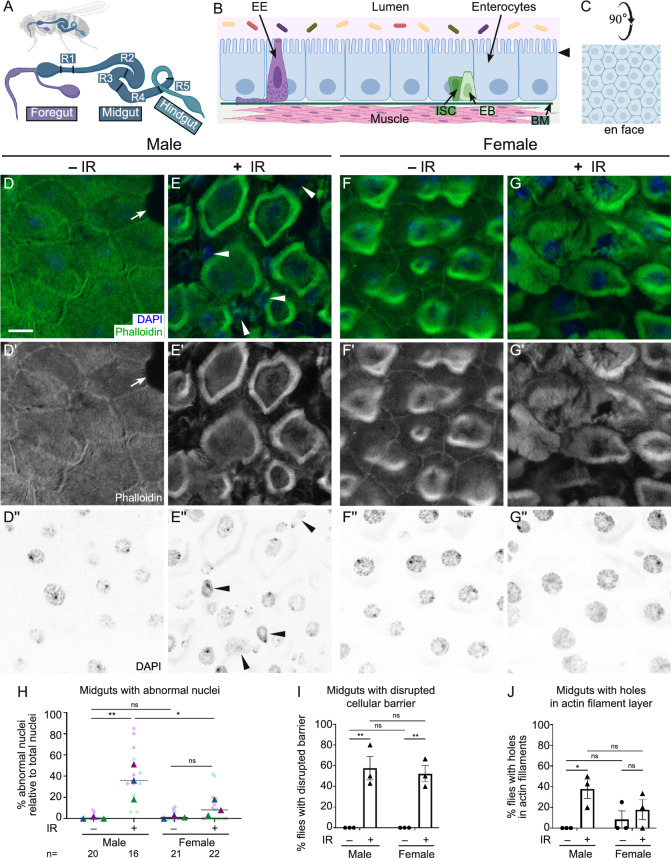
*Drosophila* male guts are more sensitive to the effects of irradiation compared to females. (**A**,**B**) Schematics of adult *Drosophila* gut structure. (**A**) Schematic of anatomical structure of the adult gut indicating the foregut, midgut and hindgut. R = Region. (**B**) Schematic of the cell types and general composition of the midgut. *EE* enteroendocrine cells, *ISC* intestinal stem cell, *EB* enteroblast, *BM* basement membrane. Arrowhead indicates the focal plane for **D**–**G**″. (**C**) General schematic of B rotated 90° to represent the en face view for **D**–**G**″. (**D**–**G**″) Dissected midguts from 4-day-old adults exposed to IR (+ IR) at day 2 and unexposed controls (− IR) labeled with phalloidin to label actin filaments and DAPI to label nuclei. The images of the R4 regions of midguts from each experimental condition were obtained by confocal microscopy using z-stacked images to show the layer of enterocytes. (**D**–**D**″) The enterocytes of a representative non-irradiated male control showing clear cellular barriers (**D**,**D**′) and good shape of nuclei (**D**,**D**″). Arrows in **D**,**D**′ indicate normal cellular gaps as visualized by phalloidin potentially due to erebosis that we did not include in our analysis^58^. (**E**–**E**″) The enterocytes of a representative male two days after irradiation showing abnormal nuclear shape (**E**,**E**″, arrowheads) and ambiguous cellular morphology due to disrupted cellular barriers (**E**,**E**′). (**F**–**F**″) The enterocytes of a representative female control and (**G**–**G**″) a representative female two days after irradiation showing normal nuclear morphology (**F**,**F**″,**G**,**G**″) compared to irradiated males (**H**). Irradiated females had disrupted cellular barriers between enterocytes (**G**,**G**′,**I**) similar to irradiated males (**E**,**E**′,**I**). (**H**) Graph indicates the percentage of abnormal nuclei relative to total nuclei within each image of the guts from irradiated or non-irradiated males and females in (**A**–**D**″). The triangles represent the arithmetic mean of each replicate. The dots represent the individual images analyzed in each replicate. The colors are: Replicate 1: dark blue triangle, light blue dots; Replicate 2: dark purple triangles, light purple dots: Replicate 3; dark green triangle, light green dots. The following numbers of the midguts were assayed per experiment: male (− IR) (6, 7, 7); male (+ IR) (5, 7, 4); female (− IR) (4, 8, 9); female (+ IR) (5, 8, 9). n: total image numbers analyzed. (**I**,**J**) Each graph indicates the percentage of animals with guts showing disrupted cellular barriers (**I**) and holes in the actin filament layer in enterocytes (**J**). Each point on the graph represents the percentage of each replicate, and each bar represents the arithmetic mean of triplicates with a standard error of the mean (SEM). The quantification method is further described in the Materials and Methods section. Data were analyzed statistically using a two-way ANOVA with multiple comparisons, followed by Tukey’s post hoc analysis. Significance of multiple comparisons: (**E**) p = 0.0077 (**) for males and 0.0384 (*) for male (+ IR) vs female (+ IR); (F) p = 0.0016 (**) for males and 0.0030 (**) for females; (**G**) p = 0.0379 (*) for males. *ns* not significant. (**D**,**E**,**F**,**G**) Green = phalloidin, blue = DAPI. (**D**′,**E**′,**F**′,**G**′) White = phalloidin. (**D**″,**E**″,**F**″,**G**″) White = DAPI. (**D**–**J**) − IR: non-irradiated, + IR: 1000 Gy irradiation. Scale bar: 10 μm in **D** for (**D**–**G**″).

As a single-layered epithelium, the adult fly midgut responds quickly to environmental changes, including nutrient fluctuations and tissue damage, in order to maintain homeostasis. Given this rapid physiological response, we conducted immunostaining on dissected midguts from flies exposed to 1000 Gy to identify physiological differences between males and females (Fig. 2, Fig. S1). The *Drosophila* adult gut is comprised of the foregut, midgut, and hindgut (Fig. 2A)^24,26^. The midgut is divided into five regions (R1-5) primarily by cellular function^40^. R4 is commonly studied due to its high incident of intestinal stem cells (ISCs), high cellular turnover, and high expression of stress-responsive genes, thus making it a good region to examine potential tissue damage from irradiation^22,23,25^. Enterocytes comprise ~ 80% of gut cells and are large, polyploid and in a single epithelial later (Fig. 2B,C)^23,41^. Other cell types, including ISCs, enteroblasts and enteroendocrine cells are less frequent, smaller and diploid (Fig. 2B). To examine potential irradiation-induced damage to the gut epithelium, we labeled the midgut enterocytes to visualize actin filaments and nuclei, then imaged the R4 region of the midguts using confocal microscopy. Irradiated males had abnormal nuclear shape (Fig. 2E,E”, H, Fig. S1D,D″,E,E″,F,F″, arrowheads), disrupted cellular barriers (Fig. 2E,E’,I, Fig. S1D,D′,E,E′), or visible holes within actin alignments (Fig. 2J, Fig. S1D,D′,E,E′, asterisks) compared to non-irradiated males that had normal nuclear shape (Fig. 2D,D″,H, Fig. S1A,A″,B,B″,C,C″) and well-organized actin filament distribution (Fig. 2D,D’,J, Fig. S1A,A′,B,B′,C,C′). In contrast, irradiated females had normal nuclear shape (Fig. 2G,G″,H, Fig. S1J,J″,K,K″,L,L″) and fewer holes within the actin cytoskeleton (Fig. 2G,G′,J) compared to irradiated males. However, irradiated females had significant numbers of enterocytes with indistinct cellular barriers compared to non-irradiated females (Fig. 2G,G′,I, Fig. S1J,J′,K,K′,L,L′). This suggests that the enterocytes of male guts are more vulnerable to the effects of IR compared to female guts, which could contribute to the difference between male and female survival after irradiation.

### *Rhodotorula taiwanensis* and* Aureobasidium pullulans* are highly radioresistant fungi

**Fig. 3:**
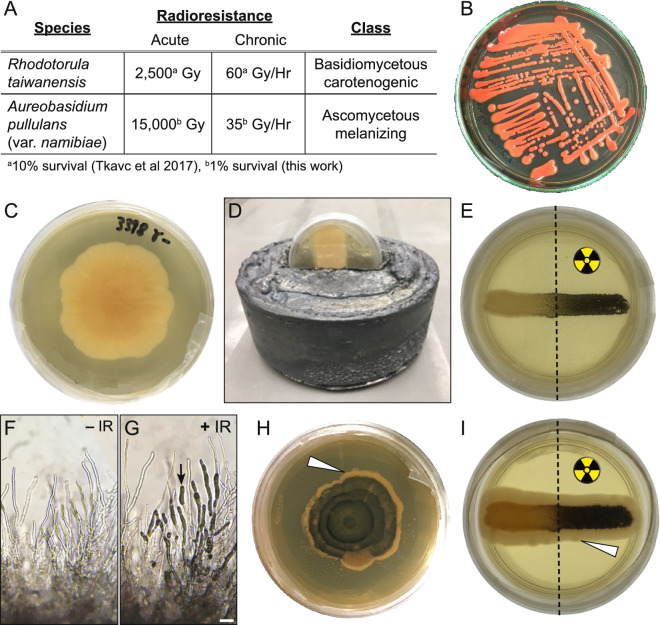
*Rhodotorula taiwanensis* and *Aureobasidium pullulans* are highly radioresistant fungi. (**A**) Characteristics of *R. taiwanensis* and *A. pullulans*. Both are highly radioresistant. (**B**) Agar plate showing streaked red *R. taiwanensis.* (**C**) Agar plate with a colony of white *A. pullulans.* (**D**) Lead shield used for irradiation experiments on an agar plate. Only the top half of the plate is exposed to irradiation. (**E**) Culture streak of *A. pullulans* protected (left) and unprotected (right) from exposure to irradiation. The right half of the culture is black due to melanin production following irradiation. (**F**,**G**) Micrographs of *A. pullulans* hyphae without (**F**, − IR) and with (**G**, + IR) irradiation. Melanin deposits (arrow) are visible in the hyphae. (**H**,**I**) Agar plate (**H**) and colony streak (**I**) of radiation-induced melanized *A. pullulans* allowed to grow without radiation*.* New fungal growth after irradiation is non-melanized and white (arrow heads). The colony shown in I is the same as the colony in E. Scale bar: 50 μm in (**G**) for (**F**,**G**).

*R. taiwanensis* and *A. pullulans* (var. *namibiae*) are highly radioresistant fungi and potential candidates for a dietary prevention strategy to injury from acute IR (Fig. 3A)^5,42^. *R. taiwanensis*, a basidiomycetous carotenogenic fungus, is radioresistant to acute exposures of 2500 Gy and chronic exposures of 60 Gy/h (Fig. 3A,B^5^). *A. pullulans*, a dimorphic ascomycetous fungus, normally grows as a white colony (Fig. 3C). We assessed that *A. pullulans* is radioresistant to acute exposures as high as 15,000 Gy and chronic exposures of 35 Gy/h (Fig. 3A,C). In response to stress such as IR, *A. pullulans* turns black due to the production of melanin^43^. Exposing a partially lead-shielded *A. pullulans* plated culture to IR caused the exposed half to produce melanin and turn black (Fig. 3D, E). IR-exposed hyphae accumulated melanin in the hyphal tips (Fig. 3F vs G, arrow). The black, melanized culture regenerated over time, with newly formed hyphae from fully exposed or shielded colonies growing white, indicating the non-melanized form replenished growth (Fig. 3H,I, arrowheads).

### *Drosophila* tolerate a diet of highly radioresistant fungi

**Fig. 4:**
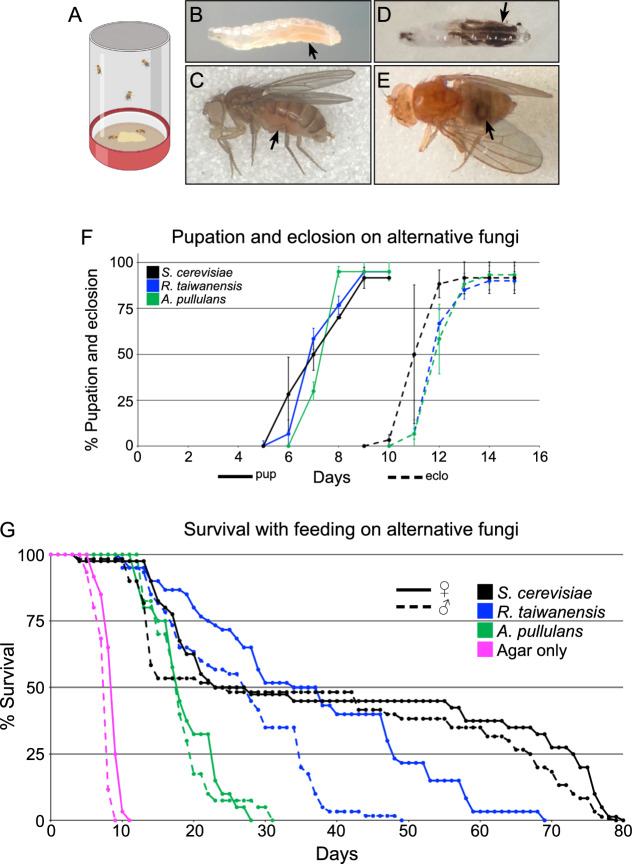
*Drosophila* tolerate dietary *Aureobasidium pullulans* and *Rhodotorula taiwanensis.* (**A**) Schematic of egg laying cups. Harvested *R. taiwanensis* and *A. pullulans* were placed on an agar plate for fly consumption (bottom). (**B**,**C**) Larva (**B**) and adult female fly (**C**) after feeding on *R. taiwanensis.* Red guts (arrows) indicate the animals ingested the fungi*.* (**D**,**E**) Larva (**D**) and adult female fly (**E**) after feeding on irradiated *A. pullulans.* Guts show the animals ingested the radiation-induced melanized fungi (arrows). (**F**) The percentage of larvae that pupated (solid lines) and eclosed (dashed lines) after being fed exclusively on alternative fungi. Each point on the graph represents the average of triplicates. Error bars represent standard deviation. (**G**) Lifespan analysis of males and females exclusively fed *A. pullulans* or *R. taiwanensis*. Agar only plates had no corn syrup (pink lines). Graphs were plotted using Microsoft Excel. Each point on the lifespan represents the average of triplicates of twenty flies that were fed fungi in parallel.

The preferred diet of *Drosophila* species is yeast growing on various organic matter, including fruit^44,45^. In a lab setting, *Drosophila melanogaster* prefers *Saccharomyces cerevisiae* (*S. cerevisiae*). To determine whether radiation-resistant fungi are a preventative dietary approach against IR, we first tested whether *Drosophila* tolerated a diet of exclusive *R. taiwanensis* or *A. pullulans* (Fig. 4). To feed flies both fungal strains, we harvested the fungi and placed a smear on a small agar plate. Flies fed ad libitum on the fungi while placed in a standard egg-laying cup with the plate on the bottom (Fig. 4A). Both larvae and adult flies ingested *R. taiwanensis* as indicated by their red guts (Fig. 4B,C, arrows). To better visualize ingested *A. pullulans*, we fed the flies irradiated, melanized fungi. The black fungi could also be seen in the larval and adult fly guts (Fig. 4D,E, arrows). This indicated that the flies readily consumed both fungal strains. We also examined the sensitivity of flies at developmental stages to determine whether fungal feeding affects pupation and eclosion rates (Fig. 4F). All larvae developed normally into pupae and eclosed at the same rate compared to control (*S. cerevisiae*) regardless of the kind of fungus (Fig. 4F). In addition, the flies survived for weeks exclusively eating either fungus (Fig. 4G). Flies fed on both strains had a reduced lifespan compared to those fed exclusive *S. cerevisiae* (Fig. 4G, blue and green vs black lines), with *A. pullulans* having a greater effect (Fig. 4G). However, neither was acutely toxic, with flies able to survive approximately 30 or 50 days, respectively, for *A. pullulans* or *R. taiwanensis*.

### Feeding *A. pullulans*, but not *R. taiwanensis*, improves male survivorship after acute irradiation

**Fig. 5:**
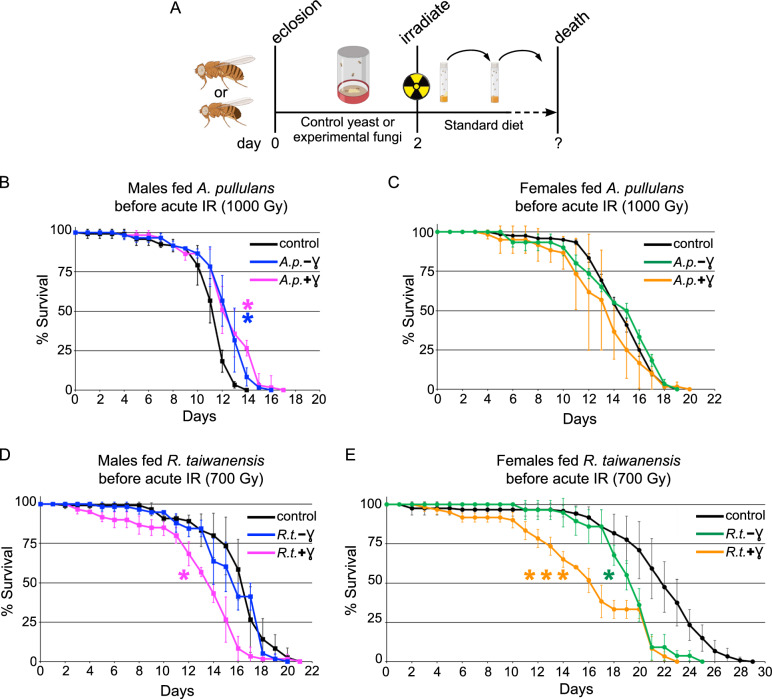
Feeding *Aureobasidium pullulans* improves lifespan in males. (**A**) Schematic showing the experimental timeline. Newly eclosed males and females are fed control yeast or experimental fungal paste for two days. The flies were subsequently placed in standard food vials and irradiated. Flies were transferred to fresh vials every 1–2 days until all died. (**B**) Lifespan of males fed non-irradiated *A. pullulans* (*A.p.*) (blue, − ɣ) and chronically irradiated *A. pullulans* (magenta, + ɣ) before exposure to 1000 Gy acute irradiation. (**C**) Lifespan of females fed non-irradiated *A. pullulans* (green, − ɣ) and chronically irradiated *A. pullulans* (orange, + ɣ) before exposure to 1000 Gy acute irradiation. Prophylactic dietary *A. pullulans* significantly extended the lifespan of males, but not females. (**D**) Lifespan of males fed non-irradiated *R. taiwanensis* (*R.t.*) (blue, − ɣ) and chronically irradiated *R. taiwanensis* (magenta, + ɣ) before exposure to 700 Gy acute irradiation. Feeding irradiated *R. taiwanensis* was detrimental to male lifespan. (**E**) Lifespan of females fed non-irradiated *R.t*. (green, − ɣ) and chronically irradiated *R. taiwanensis* (orange, + ɣ) before exposure to 700 Gy acute irradiation. Feeding non-irradiated and irradiated *R. taiwanensis* to females significantly decreased their lifespan. Graphs were plotted using Microsoft Excel. Each point on the lifespan represents the average of triplicates of twenty flies that were irradiated simultaneously. Error bars represent standard deviation. Statistical analysis was calculated using Online Application for Survival Analysis 2 (OASIS) and statistical significance was calculated using the Wilcoxon-Breslow-Gehan test. P values vs control are as follows: (**B**) p = 0.0331 (*) for *A.p.*(+ ɣ), 0.0297 (*) for *A.p.*(− ɣ) ; (**C**) p = 0.461 for *A.p.*(+ ɣ), 1.0 for *A.p.*(− ɣ) (**D**) p = 0.0041 (*) for *R.t.*(+ ɣ), 1.0 for *R.t.*(− ɣ) (**E**) p = 0.0002 (***) for *R.t.*(+ ɣ), 0.0116 (*) for *R.t.*(− ɣ).

Previous research suggested that radiation-induced metabolic changes or antioxidant production may endow fungi with radioresistance^12,46,47^. To test whether pre-feeding *Drosophila* radioresistant fungus could protect fly lifespan after acute IR exposure, we fed newly eclosed males or female for two days before exposure to acute IR, then monitored the flies for their entire lifespan (Fig. 5A). We found that flies fed *A. pullulans* showed a sex-specific improvement to lifespan, with males having increased lifespan, but females not (Fig. 5B, blue, C, green). In contrast, neither males nor females had increased lifespan after irradiation with *R. taiwanensis* pre-feeding (Fig. 5D, blue, E, green). Consistent with the exclusive diet of *R. taiwanensis* (Fig. 4G), females exhibited decreased lifespan (Fig. 5E, green). Since fungal exposure to IR upregulates protective cellular components^6–9^, we next tested whether IR-exposed, melanized fungi conferred greater dietary protection against acute IR. For *A. pullulans*, we found that males still exhibited lifespan improvement (Fig. 5B, p = 0.0331 (+ γ, magenta) vs p = 0.0297 (− γ, blue)), but this effect was not enhanced with the melanized fungus. Females again did not show any improvement in lifespan by feeding melanized *A. pullulans* (Fig. 5C). Feeding irradiated *R. taiwanensis* decreased lifespan in males and females and thus appeared to make the fungus more toxic (Fig. 5D (+ γ, magenta),E (+ γ, orange)).

### Feeding *A. pullulans* is associated with reduced IR-induced nuclear morphology defects in male guts

**Fig. 6:**
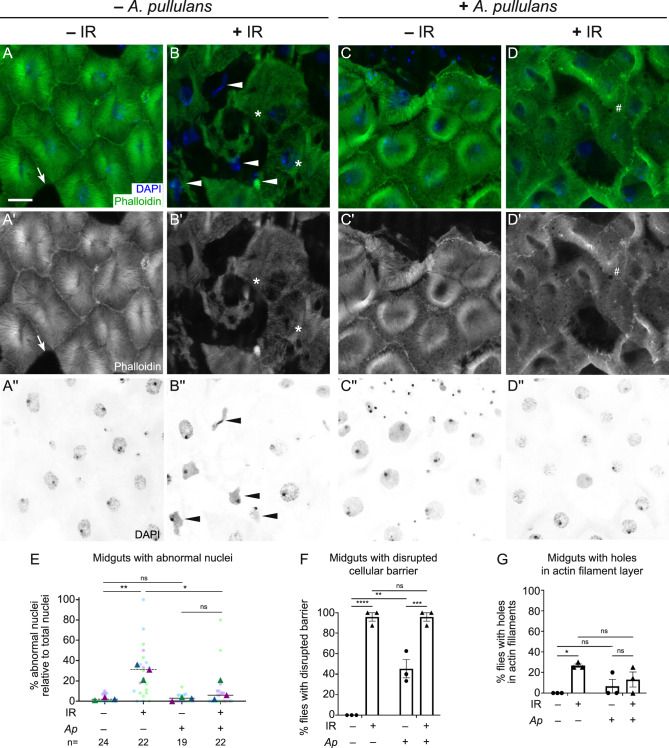
Feeding *Aureobasidium pullulans* attenuates IR-induced cellular damage in male guts. (**A**–**D**″) Dissected midguts from 4-day-old adult males exposed to IR at day 2 and control males labeled with phalloidin to label actin filaments and DAPI to label nuclei. The images of the R4 regions of midguts from each experimental condition were obtained by confocal microscopy using z-stacked images to show a layer of enterocytes. (**A**–**A**″) Representative gut dissected from a male control, showing clear cell barriers (**A**,**A**′) and normal nuclear shapes in enterocytes (**A**,**A**″). Arrows in **A**,**A**′ indicate normal cellular gaps as visualized by phalloidin potentially due to erebosis that we did not include in our analysis^58^ (**B**–**B**″) Representative gut dissected from a male two days after irradiation. Enterocytes had aberrant nuclear shape (**B**,**B**″, arrowheads, **E**) and altered cellular morphology (**B**,**B**′,**F**). Holes were formed within a layer of actin filament (**B**,**B**′, asterisk, **G**) by irradiation. (**C**–**C**″) Representative gut from a male fed *A. pullulans* (*Ap*) had normal nuclear morphology (**C**,**C**″,**E**) in enterocytes while having a loss of cell barriers with some degree (**C**,**C**′,**F**). (**D**–**D**″) Representative gut from a male fed *A. pullulans* followed by irradiation had normal nuclear shape (**D**,**D**″,**E**) as well as loss of cellular barriers and irregular cell shape (**D**,**D**′,**F**). Many small holes appeared within actin layers (**D**,**D**′, number sign) but were not counted because they did not satisfy the criterion as described in Methods. (**E**) Graph indicates the percentage of abnormal nuclei relative to total nuclei within each image of the guts from irradiated or non-irradiated males with or without *A.p.* pre-feeding. The triangles represent the arithmetic mean of each replicate. The dots represent the individual images analyzed in each replicate. The colors are: Replicate 1: dark blue triangle, light blue dots; Replicate 2: dark purple triangles, light purple dots: Replicate 3; dark green triangle, light green dots. The following numbers of the midguts were assayed per experiment: male control (11, 6, 7); male + IR (8, 4, 10); male fed *A.p.* (8, 6, 5); male fed *A.p.*-IR (7, 8, 7). n: total image numbers analyzed. (**F**,**G**) Each graph indicates the percentage of animals having guts with disrupted cellular barriers (**F**), or holes in the actin filaments layer in enterocytes (**G**). Each point on the graph represents the percentage of each replicate, and each bar presents the arithmetic mean of triplicates with a standard error of the mean (SEM). Quantification method is described in the Materials and Methods section. Data were analyzed statistically using a two-way ANOVA with multiple comparisons, followed by Tukey’s post hoc analysis. *P* values of multiple comparisons are as follows: (**E**) p = 0.0039 (**) for (−/+IR) without *A.p.* and 0.0235 (*) for (−/+*A.p.*) with IR; (F) p < 0.0001 (****) for (−/+IR) without *A.p.*, 0.0007 (***) for (−/+IR) with *A.p.*, and 0.0014 (**) for (−/+*A.p.*) without IR; (**G**) p = 0.0225 (*) for (−/+IR) without *A.p*. *ns* not significant. (**A**–**D**) Green = phalloidin, blue = DAPI. (**A**′,**B**′,**C**′,**D**′) White = phalloidin. (**A**″,**B**″,**C**″,**D**″) White = DAPI. (**A**–**G**) − IR: non-irradiated, + IR: 1000 Gy irradiation. (**E**–**G**) − *A.p.*: non-feeding *A. pullulans*, + *Ap*: feeding *A. pullulans*. Scale bar: 10 μm in **A** for **A**–**D**″.

Since male guts were more susceptible to IR exposure (Fig. 2) and pre-feeding with *A. pullulans* improved male lifespan (Fig. 5B), we examined whether *A. pullulans* pre-feeding could ameliorate any negative effects of IR on gut morphology (Fig. 6). First, we assessed the effect of feeding *A. pullulans* on gut morphology without IR exposure. Feeding male fruit flies *A. pullulans* appeared to have a dual effect on their gut enterocytes. While it reduced the number of enterocytes with abnormal nuclei (Fig. 6C,C″,E, Fig. S2G,G″,H,H″,I,I″), *A. pullulans* feeding without radiation disrupted the cells’ actin cytoskeleton**,** leading to compromised cellular barriers and altered cell shape (Fig. 6C,C′,F, Fig. S2G,G′H,H′,I,I′). This disruption might explain the observed shortened lifespan (Fig. 4G). However, when the *A. pullulans*-fed flies were exposed to radiation, they exhibited fewer abnormal nuclei in their enterocytes compared to flies that were irradiated without dietary *A. pullulans* (Fig. 6B, B″ vs D,D″,E). Even though radiation still increased the disruption of cell barriers in the *A. pullulans*-fed group (Fig. 6D,D′,F, SFig. 2J,J′,K,K′,L,L′), these findings collectively support that feeding male *Drosophila* the radioresistant *A. pullulans* helped lessen or postpone gut injury which could contribute to their improved survival following radiation exposure.

## Discussion

### *Drosophila* as a model to study dietary protection from IR

*Drosophila* has many advantages for evaluating preventative measures against radiation damage compared to mammalian models, such as experimental numbers in the hundreds, longitudinal analyses to capture survival, developmental, reproductive, and aging metrics, as well as observations of delayed effects of both radiation injury and treatment. Since irradiators can vary sample exposures due to differences in source and geometry, we created the survival curves for male and female lifespan using our ^137^Cs source, ensuring the flies were at a consistent and correct position in the irradiator and that six vials of adults, three experimental and three control, could be simultaneously exposed. As demonstrated in many species, we found sex-specific IR sensitivity in *Drosophila*, with males being more sensitive than females and thus having shorter lifespans^31–33,48^. This sex-specific difference occurred at all doses we tested. In general, female flies are more stress-tolerant than males^49–51^.

The survival radiosensitivity we observed with males compared to females was reflected in their gut morphologies, with males experiencing more damage. The GI tract is highly conserved in function and structure between *Drosophila* and vertebrates^52–54^. The fly intestinal epithelium has become a model to study tissue repair and age-related decline of tissue regeneration, including response to IR exposure^24,28,55^. The gut epithelium maintains homeostasis via normally quiescent Intestinal Stem Cells (ISCs) that rapidly proliferate in response to oxidative stress^55^. Previous studies have established that oxidative stressors, including aging, radiation, and ROS-generating compounds, as well as mitochondrial disruption, induce epithelial dysplasia and barrier dysfunction in *Drosophila*^28,56,57^. Notably, IR doses lower than those employed in the present study (2 Gy^27^; 100 Gy^28^) are sufficient to cause DSBs in the *Drosophila* midgut. Given that polyploid enterocytes have increased nuclear DNA content, which has been associated with heightened susceptibility to DSBs^16^, we hypothesize that enterocyte IR damage to the nucleus is exacerbated. The lifespan rescue observed with *A. pullulans* pre-feeding coincides with reduced IR-induced nuclear morphology deficits, suggesting a potential link between these two phenotypes. This could result in preserved gut function contributing to extended longevity. Although previous work demonstrated that 100 Gy exposure increases both DSBs and gut barrier dysfunction^28^, our findings did not show an improvement in cellular barrier integrity in irradiated males following *A. pullulans* feeding. Furthermore, cellular barrier integrity seemed to be the most sensitive of the phenotypes we scored since this was the only disruption that we observed with irradiated females. In addition, we observed increased barrier disruption with *A. pullulans* pre-feeding alone in males (Fig. 6F), suggesting that this specific diet may induce a mild stress response when given in isolation and supporting that disruption to the cellular barrier integrity may be particularly sensitive to perturbation. For assessing cellular barrier defects, we intentionally excluded cells exhibiting a loss of actin labeling and faint DAPI staining from the disrupted category. This exclusion was based on the observation of this phenomenon in non-irradiated control flies, suggesting it could be due to erebosis—a natural, non-pathological cell death process in the healthy gut^58^—rather than IR-induced pathology; further marker analysis was not performed to confirm this. Relatedly, pre-feeding *R. taiwanensis* shortened lifespan. Consistent with these complex microbial interactions, pre-feeding with unirradiated (for females) and irradiated (for males and females) *R. taiwanensis* shortened the lifespan of flies. We speculate that this detrimental effect may stem from IR exposure increasing the production of toxic compounds by *R. taiwanensis* or leading to excessive gut overpopulation in IR-compromised hosts. Interestingly, while both *R. taiwanensis* and *A. pullulans* reduced lifespan compared to the preferred control fungus, *S. cerevisiae*, the exclusive *R. taiwanensis* diet resulted in longer lifespans than the exclusive *A. pullulans* diet. Despite the lack of observed barrier improvement, the finding that *A. pullulans* pre-feeding improved male gut nuclei morphology after IR exposure we believe is significant. Given the high degree of conservation in gastrointestinal (GI) architecture and signaling pathways between *Drosophila* and mammals, dietary *A. pullulans* represents a promising nutritional preventative measure that could directly protect the GI tract against IR-induced injury.

### Mechanisms and molecules for IR protection

Ionizing radiation (IR) causes cellular injury through both direct and indirect mechanisms. Direct damage involves IR-induced modifications to DNA, proteins, and lipids, including critical DNA repair enzymes. The primary indirect mechanism is water radiolysis, which increases the concentration of reactive free radicals that subsequently disrupt cellular integrity. The differential IR tolerance observed across Metazoan species is hypothesized to stem from a combination of superior DNA protection and an enhanced capacity to manage reactive oxygen species (ROS)^59^. Similarly, the highly radioresistant fungal pathogen, *Cryptococcus neoformans*, employs dual radioprotection strategies: a robust enzymatic antioxidant system alongside upregulated DNA damage repair genes^47,60^. If *A. pullulans* utilizes analogous defensive strategies, it is difficult to postulate a clear pathway for the transport of these fungal enzymes into host enterocytes to confer radioprotection. Nevertheless, compelling evidence suggests that the natural human microbiome mitigates ROS in enterocytes by secreting synthesized metabolites and upregulating host antioxidant enzymes within gut cells^61^. How might *A. pullulans* exert its protective effect on the gut? One possibility is that ingested antioxidant metabolites or other small molecules from *A. pullulans* could be taken up by the enterocytes, thus increasing protection against free radicals. For example, feeding mice melanin-containing Jelly Ear mushrooms compared to porcini mushrooms greatly improved survival post 9 Gy IR, with no signs of GI radiation damage 45 days post-irradiation^62^. Survival improvement post-irradiation has also been demonstrated with the oral administration of antioxidants^63,64^. *N*-acetyl-l-cysteine as a dietary supplement protected the mouse intestinal epithelial barrier after irradiation^65^. Mice ingesting *Aloe vera* extract had delayed radiation sickness symptoms and lower acid phosphatase and alkaline phosphatase levels in the liver after 6 Gy irradiation compared to a control^66^. A mixture of ingested traditional Indian medicinal plants increased survival and improved DNA damage compared to a control after 7.5 Gy exposure^67^. In *Drosophila*, feeding curcumin, a plant phenolic compound, increased lifespan and decreased protein carbonylation^68^. Feeding flies a tea polyphenol and beta-carotene decreased mutation frequency and increased antioxidant levels post 10 Gy exposure compared to controls^69^. Finally, flies fed ibuprofen or two flavonoids (quercetin and epicatechin) showed increased lifespan compared to a control after 1000 Gy exposure^70^.

Pre-feeding MnCl_2_ before IR exposure increased radiation survival in a similar manner to *A. pullulans* feeding^71^. The protective effect of dietary manganese was attributed to an increase in small molecule manganous peptide antioxidant content, not an increase in the free radical scavenger Mn Superoxide Dismutase. It is possible that *A. pullulans* contains high levels of manganous peptides, and this could explain its protective effect. Melanin is recognized as a radioprotectant present in extremophile microorganisms and has been shown to have radioprotective effects in vertebrates as well^62,72,73^. Mice fed melanin-containing mushrooms had greater gastrointestinal protection compared to mice fed non-melanized mushrooms, supporting that the presence of melanin in the gut could protect the gut lining from the effects of acute irradiation^62^. *A. pullulans* melanizes in response to IR^10^. Since this fungus extended lifespan in irradiated males, we also tested the melanized phenotype to determine whether melanin could increase its effectiveness. However, melanized *A. pullulans* did not increase the protective effect, suggesting that the concentration of melanin was not sufficient to produce an enhanced radioprotective effect or that other metabolites or molecules are responsible for the protection. Finally, we cannot rule out that the flies pre-fed *A. pullulans* before transfer to a standard diet supplemented with dry yeast eat less *A. pullulans* since it is not their preferred food source. Dietary restriction (DR) extends lifespan in many different organisms with female fruit flies responding more robustly compared to male flies^74,75^. Mair et al. demonstrated that for female fruit flies, this was independent of calories and that restricting yeast (protein) only while maintaining total calories with sugar mimics the lifespan extension observed with DR^76^. Examining mortality rates with DR, Mair et al. found that switching males from fully fed to DR reduced mortality, but switching them from DR to fully fed increased mortality rates^77^. Likewise, switching males from a low calorie to a high calorie diet decreased lifespan^78^. We would posit that males fed *A. pullulans* for two days before standard food would most likely mimic DR before switching to fully fed conditions. For this reason, it does not seem likely that two days of *A. pullulans* pre-feeding would be sufficient to increase lifespan in males. In addition, we did not observe any change in lifespan for females which are more responsive to DR.

### Wider implications of identifying dietary strategies for reducing radiation-induced cellular damage

Radiotherapy patients are the most recognizable cohort that would benefit from identifying new strategies for reducing the effects of radiation injury. Radiotherapy patients, particularly those exposed to abdominal and pelvic radiation therapy, frequently have the side effect of GI tract damage and enteropathy^79,80^. An important consideration is that symptoms may be delayed and impact long-term quality of life. In addition to tissue damage, one notable change in this cohort is the alteration in the microbiome^81^. Changes to metabolites, tryptophan pathway intermediates in particular, and the microbiome occur in mice that exhibit improved survivorship to irradiation as well as in patients with leukemia who experienced fewer adverse effects of whole-body irradiation^82^. Identifying protective measures would greatly aid these patients.

An additional population at serious health risk from IR are those who are potentially exposed to radiation disasters—either as cleanup workers, military personnel, or the general public. For these groups of people, the diverse and wide-ranging effects of IR exposures are organized into subsyndromes of acute radiation syndrome (ARS). While medical countermeasures to the hematopoietic subsyndrome of acute radiation syndrome (H-ARS) have been developed, no medical countermeasures to the gastrointestinal subsyndrome (GI-ARS) exist. There are only four countermeasures approved as mitigators by the FDA for H-ARS, but none are effective as prophylactic intervention or for GI damage^83^. Gamma tocotrienol, a naturally occurring vitamin E isoform, reportedly confers some radioprotective survival in mice^64,84,85^. Effective prophylactic countermeasures targeting the vulnerable gastrointestinal tract need to be identified^85,86^.

### Future directions and limitations

This study identified *A. pullulans* as a dietary prevention strategy for protection against acute irradiation. However, this observation has certain limitations and opens several avenues for future research. A key limitation is that we have only observed protection when feeding the entire fungus; therefore, a future direction is to identify the precise compound(s) responsible for this effect. While some contributors to the fungus’s radioresistance have been identified, such as manganous peptides, it is likely that other, as-yet-unidentified mechanisms and metabolites are also involved. In addition, we only tested two days of pre-feeding with *A. pullulans* for radioprotection. Future studies could extend this analysis to feeding for longer or to supplement standard food in various combination with live or heat inactivated or irradiated and non-irradiated *A. pullulans* to improve the effect. Furthermore, although we demonstrated improved lifespan and gut morphology in male flies, the underlying mechanism remains to be determined. In the future, many other radioresistant fungi could be tested in a similar manner. If subsequent vertebrate studies indicate that dietary *A. pullulans* can protect against radiation injury, it could prove to be a significant benefit for humans. Employing whole fungi that have adapted to survive high doses of irradiation may be the optimal strategy to protect people from the deleterious effects of IR.

## Supplementary Information

Supplementary Figure 1.

Supplementary Figure 2.

Supplementary Table 1.

Supplementary Spreadsheet 1.

## Data Availability

The images analyzed during the current study will be made available from the corresponding author on reasonable request. All other data generated are included in this published article and the supplementary information files.
