# Supplementary material for: Feeding Drosophila highly radioresistant fungi improves survival and gut morphology following acute gamma radiation exposure
Source: Sci Rep. 2025 Dec 14;16:1855. doi: 10.1038/s41598-025-31545-6 (PMC12804729; doi:10.1038/s41598-025-31545-6)
Supplement: Supplementary file 2 — Supplementary Figure 2. [file 41598_2025_31545_MOESM2_ESM.pdf]

Figure S2. Prophylactic dietary *A. pullulans* mitigates IR-induced cellular damage in male guts

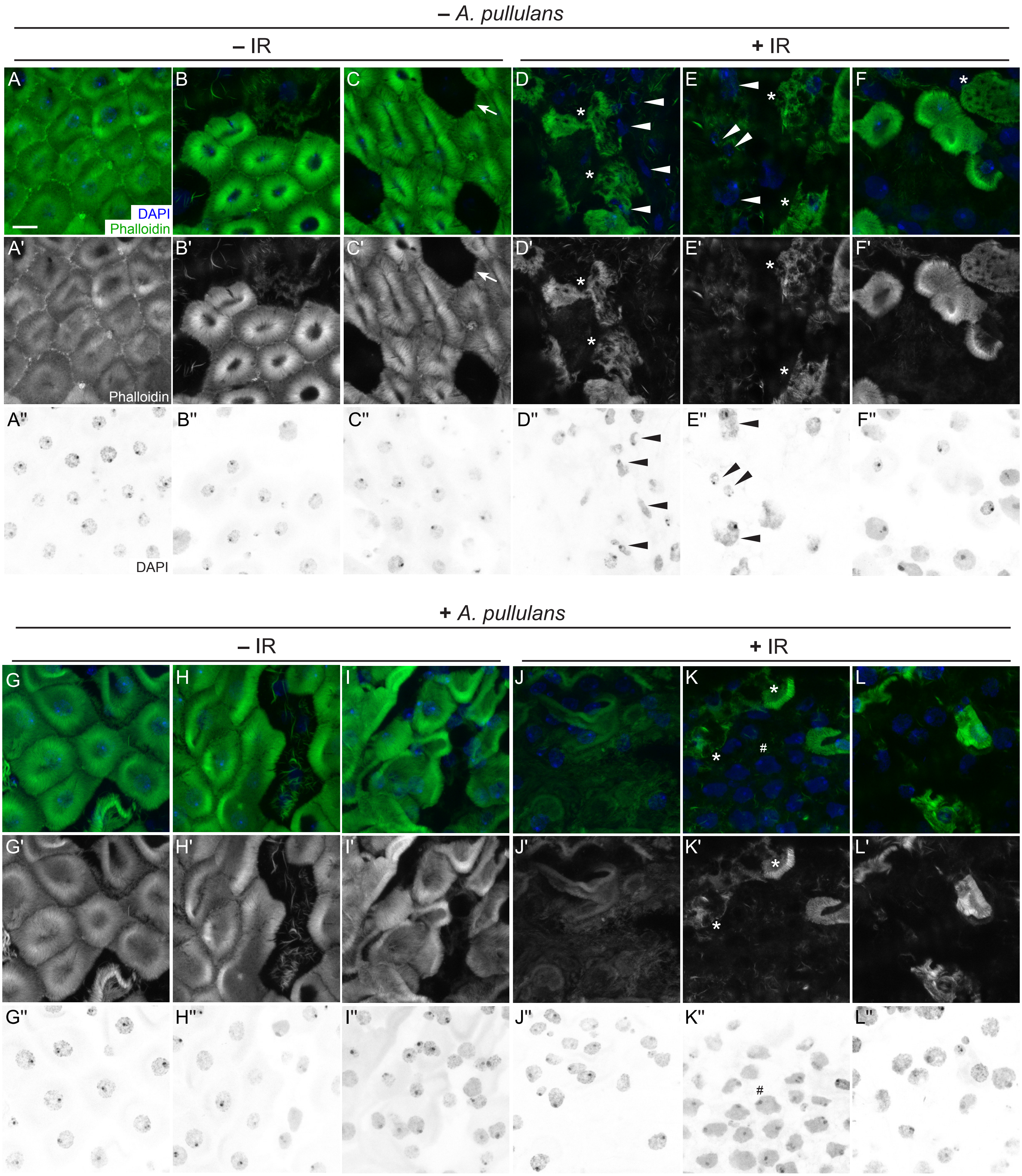

Supplemental Figure 2. Prophylactic dietary *Aureobasidium pullulans* mitigates IR-induced damaging process of male guts.

(A-C”) R4 regions of midguts from male controls. Dissected midguts were labeled with phalloidin to label actin filaments and DAPI to label nuclei and then imaged using a confocal microscope with z-stacks for a layer of enterocytes. The enterocytes had clear cellular barriers (A,A’,B,B’,C,C’) and normal nuclear shape (A,A”,B,B”,C,C”). Arrows in C, C’ indicate normal cellular gaps as visualized by phalloidin potentially due to erebosis that we did not include in our analysis. (D-F”) R4 regions of midguts from males two days after irradiation. Immunostaining shows abnormal nuclear shape (D,D”,E,E”), disrupted cellular barriers (D,D’E,E’F,F’), and holes within an actin filament layer (D,D’E,E’F,F’, asterisk). (G-I”) R4 regions of midguts from males fed *Aureobasidium pullulans* (*Ap*)***.*** *Ap* feeding caused some degrees of loss of cellular barriers (G,G’,H,H’,I,I’). (J-L”) IR following *Ap* feeding did not induce smaller nuclei although nuclear morphology was altered (K,K”, number sign). Actin filaments structure was severely changed by IR (J,J’,K,K’,L,L’). (A,B,C,D,E,F,G,H,I,J,K,L) Green = Phalloidin, blue = DAPI. (A’,B’,C’,D’,E’,F’,G’,H’,I’,J’,K’,L’) White = phalloidin. (A”,B”,C”,D”,E”,F”,G”,H”,I”,J”,K”,L”) White = DAPI. Scale bar: 10 µm in A for A-L”.
